# Cognitive reserve and everyday functioning in cognitively unimpaired older adults: evidence from the CAN-PROTECT study

**DOI:** 10.1093/gerona/glag227

**Published:** 2026-09-12

**Authors:** Maryam Ghahremani, Dylan X Guan, Clive Ballard, Byron Creese, Anne Corbett, Ellie Pickering, Eric E Smith, Zahinoor Ismail

**Affiliations:** Psychiatry, Cumming School of Medicine, University of Calgary, Calgary, Alberta, Canada; Hotchkiss Brain Institute, Cumming School of Medicine, University of Calgary, Calgary, Alberta, Canada; Hotchkiss Brain Institute, Cumming School of Medicine, University of Calgary, Calgary, Alberta, Canada; Clinical and Biomedical Sciences, Faculty of Health and Life Sciences, University of Exeter, Exeter, UK; Department of Psychology, College of Health Medicine and Life Sciences, Brunel University of London, London, UK; Clinical and Biomedical Sciences, Faculty of Health and Life Sciences, University of Exeter, Exeter, UK; Clinical and Biomedical Sciences, Faculty of Health and Life Sciences, University of Exeter, Exeter, UK; Hotchkiss Brain Institute, Cumming School of Medicine, University of Calgary, Calgary, Alberta, Canada; Clinical Neurosciences, Cumming School of Medicine, University of Calgary, Calgary, Alberta, Canada; Community Health Sciences, Cumming School of Medicine, University of Calgary, Calgary, Alberta, Canada; Psychiatry, Cumming School of Medicine, University of Calgary, Calgary, Alberta, Canada; Hotchkiss Brain Institute, Cumming School of Medicine, University of Calgary, Calgary, Alberta, Canada; Clinical and Biomedical Sciences, Faculty of Health and Life Sciences, University of Exeter, Exeter, UK; Department of Psychology, College of Health Medicine and Life Sciences, Brunel University of London, London, UK; Clinical Neurosciences, Cumming School of Medicine, University of Calgary, Calgary, Alberta, Canada; Community Health Sciences, Cumming School of Medicine, University of Calgary, Calgary, Alberta, Canada; Department of Pathology and Laboratory Medicine, University of Calgary, Calgary, Alberta, Canada; O’Brien Institute for Public Health, Cumming School of Medicine, University of Calgary, Calgary, Alberta, Canada; (Medical Sciences Section)

**Keywords:** Education, Activities of daily living, Function, Quality of life, Dementia

## Abstract

**Background:**

Cognitive reserve (CR) is thought to buffer against the clinical manifestation of dementia, preserving cognitive function despite accumulating pathology. However, less is known about how CR relates to the capacity to independently perform activities of daily living (ADL), particularly among cognitively unimpaired (CU) older adults. Here, we investigated this association.

**Methods:**

Baseline data from 1571 CU participants (mean age: 64.7 ± 7.6; 81.3% female) from the CAN-PROTECT study were analyzed. A composite CR score was derived from education, occupational complexity, and engagement in cognitively stimulating activities. Functional ability was measured using the Standard Assessment of Global Everyday Activities (SAGEA) scale, capturing cognitive, applied cognitive, instrumental, and basic ADL, and mobility. Negative binomial models examined associations between CR and global and domain-specific ADL, adjusting for age, sex, cognition, behavior, and physical/sensory limitations. Additional models tested the independent contribution of CR components to functional outcomes. Mediation analyses assessed the effect of CR on quality of life (QoL) through ADL abilities.

**Results:**

Higher CR was associated with lower global ADL impairment (count ratio = 0.58, 95% CI, 0.36-0.94, *p* = .027), driven by 49% less impairment in applied cognitive ADL (95% CI, 0.26-0.97, *p* = .038). Among CR domains, education showed the most consistent association with lower impairment across all ADL domains and was indirectly linked to better QoL through improved function.

**Conclusions:**

CR, particularly education, may play an important role in supporting functional abilities and QoL among CU older adults. Enhancing CR may be a promising avenue to promote functional independence and healthy aging, even in the absence of cognitive impairment.

## Introduction

Cognitive reserve refers to the brain’s capacity to optimize performance through differential recruitment of networks or alternative cognitive strategies, allowing some individuals to maintain cognitive function despite the presence of neuropathology.^1^ Cognitive reserve is shaped across the lifespan by education, occupational complexity, social engagement, and participation in cognitively stimulating activities.^2^ A substantial body of research indicates that higher cognitive reserve can delay the clinical manifestation of neurodegenerative changes, particularly in the early disease stages, slowing progression from normal cognition to mild cognitive impairment (MCI) and dementia.^2^^,^^3^

While the relationship between cognitive reserve and cognitive outcomes has been extensively studied, considerably less is known about how cognitive reserve is related to functional abilities, including basic activities of daily living (BADL) and instrumental activities of daily living (IADL). BADL capture fundamental physical activities such as feeding, bathing, dressing, and mobility, whereas IADL encompass managing finances, using transportation, organizing medications, preparing meals, and navigating social or community settings.^4^ IADL require the integration of multiple cognitive domains, including memory, executive function, attention, and visuospatial skills. Preservation of both BADL and IADL is essential for independent living and quality of life (QoL) in older adults. Identifying factors, such as cognitive reserve, that may protect against early functional decline could inform preventive interventions, lifestyle recommendations, and policies aimed at promoting QoL and delaying disability and institutionalization.

In dementia research, functional ability is often assessed in individuals with established cognitive impairment, with loss of independence serving as the threshold for dementia diagnosis. However, subtle impairments in complex functional abilities such as IADL may appear earlier, sometimes preceding measurable deficits on standard neuropsychological tests,^5–8^ potentially signaling underlying neurodegeneration. Understanding how cognitive reserve relates to the preservation of these complex functions in early disease stages could reveal important protective mechanisms that support independent living and delay the onset of functional disability that portends dementia.

While studies examining cognitive reserve and activities of daily living (ADL) in dementia-free populations are limited, a few have investigated this relationship. In community-dwelling older adults, years of education, a common proxy for cognitive reserve, moderated the association between global cognition and IADL,^9^ suggesting that individuals with lower education, and thus lower cognitive reserve, may be more vulnerable to functional decline. More recent work in a large MCI sample examined multiple cognitive reserve proxies, education, occupation, and cognitively stimulating leisure activities, and found that leisure engagement showed the strongest association with both BADL and IADL functioning, whereas education and occupation showed negligible relationships.^10^ These results suggest that lifestyle factors beyond formal education may be important for preserving real-world functional abilities, though the influence of different cognitive reserve proxies may also vary by disease stage. Furthermore, it remains unclear whether cognitive reserve confers similar benefits earlier along the cognitive continuum, underscoring the need to explore these relationships in cognitively unimpaired (CU) populations.

Here, we examined whether higher cognitive reserve is associated with better basic and complex ADL functioning in CU older adults. We further interrogated the association between cognitive reserve and QoL, and the potential mediating role of ADL.

## Methods

### Study population

Participant data were obtained from the July 2025 release of the Canadian Platform for Research Online to Investigate Health, Quality of Life, Cognition, Behavior, Function, and Caregiving in Aging (CAN‐PROTECT).^11^ CAN-PROTECT is an online, nationwide, longitudinal observational cohort study examining risk and resilience in brain aging in dementia‐free, community‐dwelling adults. All residents of Canada aged ≥18 years are eligible for enrollment, excluding individuals with an established diagnosis of dementia or those unable to provide informed consent. Participants and their study partners complete annual assessments covering demographics, health, cognition, behavior, function, lifestyle, and other domains. Ethics approval for this study was granted by the Conjoint Health Research Ethics Board at the University of Calgary (REB21‐1065). A detailed description of the CAN‐PROTECT study design and procedures has been published previously.^11^

### Participant selection

From the initial cohort of 3202 participants, 2338 participants had completed the neuropsychological test battery, meeting the prerequisite for access to other study components. Participants were then sequentially excluded for the following reasons: age <50 years (*n* = 278), a diagnosis of MCI (*n* = 19), incomplete lifestyle questionnaire (*n* = 177), missing data on assessments of daily function (*n* = 286), quality of life (*n* = 4), or behavior (*n* = 3), resulting in a final analytic sample of 1571 participants (Figure 1).

**Figure 1:**
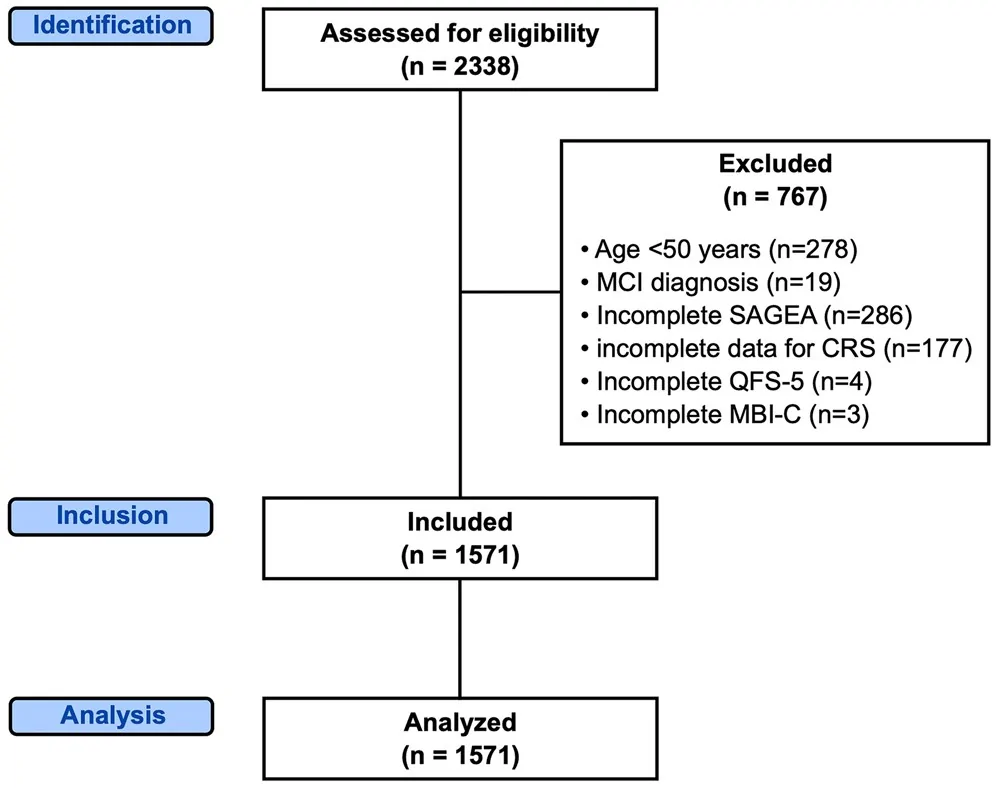
Flowchart demonstrating the participant selection process. CRS, cognitive reserve score; MBI-C, mild behavioral impairment checklist; MCI, mild cognitive impairment; QFS-5, Quality of life and function self-report; SAGEA, Standard Assessment of Global Everyday Activities.

### Measures

#### Cognitive reserve

Cognitive reserve score (CRS) was calculated based on a published algorithm^12^ using demographics and lifestyle questionnaire data, capturing cognitive reserve across 3 domains: education (2 items), occupation (5 items), and personal activities (6 items). This score has previously been shown to be associated with higher neuropsychological test performance and lower odds of subjective cognitive decline.^12^ The education domain included the highest educational level attained and total years of formal education. The occupation domain captured job type, self‐reported cognitive demands of the job, and the highest complexity level of work involving data, people, and objects.^13^ The personal activities domain encompassed musical ability (playing an instrument), multilingualism, and frequent engagement in cognitively stimulating activities such as word/number puzzles or computer‐based brain training. The total composite CRS ranged from 0 to 1, with higher scores indicating greater educational attainment, occupational complexity, or personal activity engagement.

#### Daily function

Daily function was captured using the Standard Assessment of Global Everyday Activities (SAGEA) scale,^14^ which evaluates the level of self-reported difficulties in performing ADL across 5 domains: (1) cognitive ADL (eg, maintaining attention in conversation, remembering recent events, multitasking); (2) applied cognitive ADL (eg, playing concentration-based games, navigating new environments, organizing trips or social activities); (3) IADL (eg, managing finances, medications, house chores, transportation, driving); (4) BADL (eg, dressing, bathing, toileting); and (5) mobility (eg, walking, using stairs). Each item is scored from 0 (no difficulty) to 3 (severe/stopped due to difficulty). Scores within each domain were summed to generate domain-level scores, which were then summed across all domains to yield a total score (range: 0-45), with higher scores indicating greater functional impairment.

#### Quality of life

QoL was assessed using the Quality of Life and Function Self-report (QFS-5),^15^ a novel abilities-based scale that assesses QoL and function across 5 domains: (1) productivity (eg, ability to work, manage responsibilities, attend appointments, travel, and handle finances); (2) self-care (eg, living independently, maintaining a clean home and personal hygiene, preparing food, and managing health); (3) social relationships (eg, getting along with others, maintaining close relationships, feeling part of a community); (4) life satisfaction (eg, enjoyment of life, sense of purpose, autonomy, feeling supported); and (5) leisure activities (eg, engaging in hobbies, learning for enjoyment, participating in sports/clubs, and relaxation activities). Since our study assessed function independently using the SAGEA scale, to avoid overlap with ADL items, only items across the life satisfaction and social relationships domains of QFS-5 were considered to capture QoL. Each item was rated from 0 to 3, with the total composite score obtained from the sum of scores across items within the life satisfaction and social relationships domains. Higher scores indicate better QoL.

#### Cognition

Cognitive performance was assessed using the CAN-PROTECT neuropsychological test battery, which included the following: Trail Making B (visual attention and task switching), Switching Stroop (visual attention and task switching), Self‐Ordered Search (spatial working memory), Paired Associate Learning (visual episodic memory), Verbal Reasoning (general intelligence and grammatical reasoning), and Digit Span (working memory).^11^^,^^16^^,^^17^ The total score was calculated as the sum of standardized scores across all tests (z-score), with higher scores indicating better cognitive function.

#### Behavior

Behavioral symptoms were assessed using the MBI checklist (MBI-C),^18^ which assesses later-life emergent and persistent neuropsychiatric symptoms (NPS) across 5 domains: decreased motivation, affective dysregulation, impulse dyscontrol, social inappropriateness, and abnormal perception/thought content. The MBI total score was obtained from the sum of scores across all domains, with higher scores indicating greater NPS severity.

#### Physical/sensory limitations

To help distinguish ADL difficulties secondary to underlying neurodegeneration from those arising from physical or sensory disability, we incorporated the SAGEA questionnaire item asking whether, over the past month, any of the following problems limited the participant’s ability to perform activities (yes/no): physical injury, vision loss, arthritis, unsteadiness, and chronic pain.

### Statistical analysis

All statistical analyses were performed in RStudio version 2024.09.0 + 375 (R version 4.2.2)(R Foundation for Statistical Computing, Vienna, Austria). Sample characteristics included baseline age, sex, education years, neuropsychological test scores, CRS, total and domain-level SAGEA scores, QFS-5 composite score, MBI-C total score, and presence or absence of physical/sensory limitations to perform ADL. All variables were summarized using means, standard deviations (*SD*), and ranges for continuous measures, and percentages for categorical measures.

For all regression models, the CRS and its individual domain scores were standardized to a z-score to facilitate interpretation of effect estimates. The association between CRS z-score and SAGEA total score (outcome) was modeled using negative binomial regression from the *MASS* package, given the right-skewed distribution of non-negative integer SAGEA scores with overdispersion (ie, variance > mean). Separate models were implemented to explore associations with individual ADL domains. All models adjusted for age, sex, neuropsychological test score, MBI-C total score, and physical/sensory limitations to ADL performance. While cognitive, applied cognitive, and instrumental ADL all involve cognitively demanding activities, the cognitive ADL items (ie, remembering events, maintaining attention, and task-switching) more directly parallel processes assessed by certain neuropsychological tests, potentially leading to over-adjustment when test scores are included. Accordingly, sensitivity analyses excluding the neuropsychological test score were conducted for models with cognitive ADL as the outcome.

Secondary analyses explored the association between each standardized CRS domain (education, occupation, personal activities) and SAGEA scores, including total and domain-specific scores. All results were reported as count ratios (CRs), where the exponentiated coefficients represent multiplicative differences in the expected raw outcome counts in association with 1 *SD* increase in standardized CRS. Estimates were presented with 95% confidence intervals (CI) and *p*-values.

Mediation analyses examined the association between CRS and QoL, with SAGEA score as the mediator using the *mediation* package. The analysis was conducted within a hypothesized framework in which higher cognitive reserve would be associated with better preservation of daily function, which may in turn be associated with higher QoL. The mediator model was a Poisson regression, with SAGEA score as the outcome and standardized CRS as the primary exposure, adjusted for age, sex, neuropsychological test score, MBI-C total score, and physical/sensory limitations. The outcome model was a linear regression with QFS-5 composite score as the outcome and CRS and SAGEA scores as primary exposures, adjusted for the same covariates as the mediator model. Mediation analysis was conducted using nonparametric bootstrapping with 1000 simulations. The average causal mediation effect (ACME) estimated the indirect association between CRS and QoL through ADL performance, while the average direct effect (ADE) captured the direct association between CRS and QoL, independent of ADL performance. The total effect of CRS on QoL was defined as the sum of ACME and ADE, and the proportion mediated as the ratio of ACME to the total effect. These estimates were interpreted as describing the pattern of associations among CRS, ADL, and QoL rather than confirming causal mediation effects. For all models, the statistical significance threshold was set at *p* < .05.

## Results

### Sample characteristics

The final sample of 1571 CU participants had a mean age of 64.7 ± 7.6 and 16.1 ± 5.4 years of education at baseline, with 81.3% females. The mean CRS was 0.5 ± 0.1 (range: 0.2-0.9) and mean SAGEA total score was 2.6 ± 3.3 (range: 0-27), with mean standardized neuropsychological test score of −0.1 ± 0.5, mean QFS-5 composite score of 25.6 ± 4.8, and mean MBI-C total score of 5.3 ± 7.1. Mean domain-level SAGEA scores were 1.0 ± 1.3 (range: 0-8) for cognitive-ADL, 0.8 ± 1.2 (range: 0-8) for applied-cognitive-ADL, 0.4 ± 0.9 (range: 0-8) for IADL, 0.1 ± 0.5 for BADL (range: 0-5), and 0.3 ± 0.7 (0-6) for mobility. Further, 33.7% of participants had physical/sensory limitations (Table 1). Compared to participants included in the analyses, those excluded due to missing data on lifestyle questionnaire, SAGEA, QFS-5, and MBI-C assessments (*N* = 470) had a higher proportion of males (26.0% males, 74.0% females; *p* < .001).

**Table 1:** Sample characteristics.

Variable	*N* (%)	*M* (*SD*), range
Age (years)	–	64.7 (7.6), 50-94
**Sex**		
Male	293 (18.7)	–
Female	1278 (81.3)	–
Education (years)	–	16.1 (5.4), 0-79
Neuropsychological test z-score	–	−0.1 (0.5), −1.4 to 1.6
**Cognitive reserve score**	–	0.5 (0.1), 0.2-0.9
Education	–	0.7 (0.2), 0.1-1.1
Occupation	–	0.6 (0.2), 0-1.1
Personal activities	–	0.3 (0.2), 0-0.9
**SAGEA total score**	–	2.6 (3.3), 0-27
Cognitive-ADL score	–	1.0 (1.3), 0-8
Applied-ADL score	–	0.8 (1.2), 0-8
IADL score	–	0.4 (0.9), 0-8
BADL score	–	0.1 (0.5), 0-5
Mobility score	–	0.3 (0.7), 0-6
QFS-5 composite score	–	25.6 (4.8), 3-30
MBI-C total score	–	5.3 (7.1), 0-65
**Physical/sensory limitations**		
Absent	1041 (66.3)	–
Present	530 (33.7)	–

Mean (*M*), standard deviation (*SD*), and ranges were calculated for continuous variables, while total number (*N*) and percentage (%) were calculated for categorical variables.

Abbreviations: ADL, activities of daily living; BADL, basic activities of daily living; IADL, instrumental activities of daily living; MBI-C, mild behavioral impairment checklist; QFS-5, Quality of Life and Function Self-Report; SAGEA, Standard Assessment of Global Everyday Activities.

### ADL and global cognitive reserve

Higher CRS was associated with significantly better overall functional ability. Specifically, each *SD* rise in CRS was associated with 6% lower SAGEA total scores, indicating better function (CR = 0.94, 95% CI, 0.89-0.99, *p* = .027) (Table 2). Across other model covariates, higher neuropsychological test scores were associated with lower SAGEA total scores (CR = 0.84; 95% CI, 0.74-0.95, *p* = .006), whereas higher MBI-C total scores (CR = 1.07; 95% CI, 1.06-1.08, *p* < .001) and physical/sensory limitations (CR = 1.79; 95% CI, 1.60-2.00, *p* < .001) were associated with higher total SAGEA scores, that is, poorer function.

**Table 2:** Cross-sectional associations between standardized cognitive reserve score (CRS) and global ADL performance, adjusted for age, sex, cognitive performance as z-scores on neuropsychological test battery, MBI-C total score, and physical/sensory limitations.

Exposure	CR	95% CI	*p*-Value
CRS	0.94	0.89-0.99	**.027**
Education	0.91	0.87-0.96	**.001**
Occupation	0.97	0.92-1.02	.251
Personal activities	1.01	0.95-1.06	.829

Abbreviations: ADL, activities of daily living; CI, confidence interval; CR, count ratio; MBI-C, mild behavioral impairment checklist. Bold p-values indicate statistical significance (<0.05).

Among SAGEA domains, applied cognitive ADL was the only domain that was significantly associated with CRS, where each *SD* rise in CRS was associated with 7% lower ADL scores (CR = 0.93, 95% CI, 0.86-1.00, *p* = .038). For the other domains, the direction of effect was in the same direction but the associations were not statistically significant (Cognitive ADL: CR = 0.95, 95% CI, 0.90-1.01, *p* = .116; IADL: CR = 0.95, 95% CI, 0.84-1.08, *p* = .408; BADL: CR = 0.89, 95% CI, 0.75-1.06, *p* = .171; Mobility: CR = 0.95, 95% CI, 0.83-1.08, *p* = .438) (Table 2). For the cognitive ADL domain, the association became significant in a sensitivity analysis excluding neuropsychological test scores (CR = 0.94, 95% CI, 0.89-0.99, *p* = .028).

### ADL and cognitive reserve domains

When examining individual cognitive reserve domains, higher education showed the strongest association with lower SAGEA scores across global and ADL domains (Figure 2): 9% lower global ADL (CR = 0.91, 95% CI, 0.87-0.96, *p* = .001), 6% lower cognitive ADL (CR = 0.94, 95% CI, 0.89-1.00, *p* = .046), 7% lower applied cognitive ADL (CR = 0.93, 95% CI, 0.86-1.00, *p* = .041), 11% lower IADL (CR = 0.89, 95% CI, 0.78-1.00, *p* = .055), 19% lower BADL (CR = 0.81, 95% CI, 0.69-0.96, *p* = .013), and 18% lower mobility scores (CR = 0.82, 95% CI, 0.72-0.93, *p* = .002). For the cognitive ADL domain, the association with education slightly strengthened in the sensitivity analysis excluding the neuropsychological test score from covariate adjustment (CR = 0.93, 95% CI, 0.88-0.98, *p* = .011). The occupation and personal activities domains were not significantly associated with total or domain-specific ADL scores, although occupation showed a trend toward significance with applied cognitive ADL (CR = 0.94, 95% CI, 0.87-1.01, *p* = .079) (Table 3).

**Figure 2:**
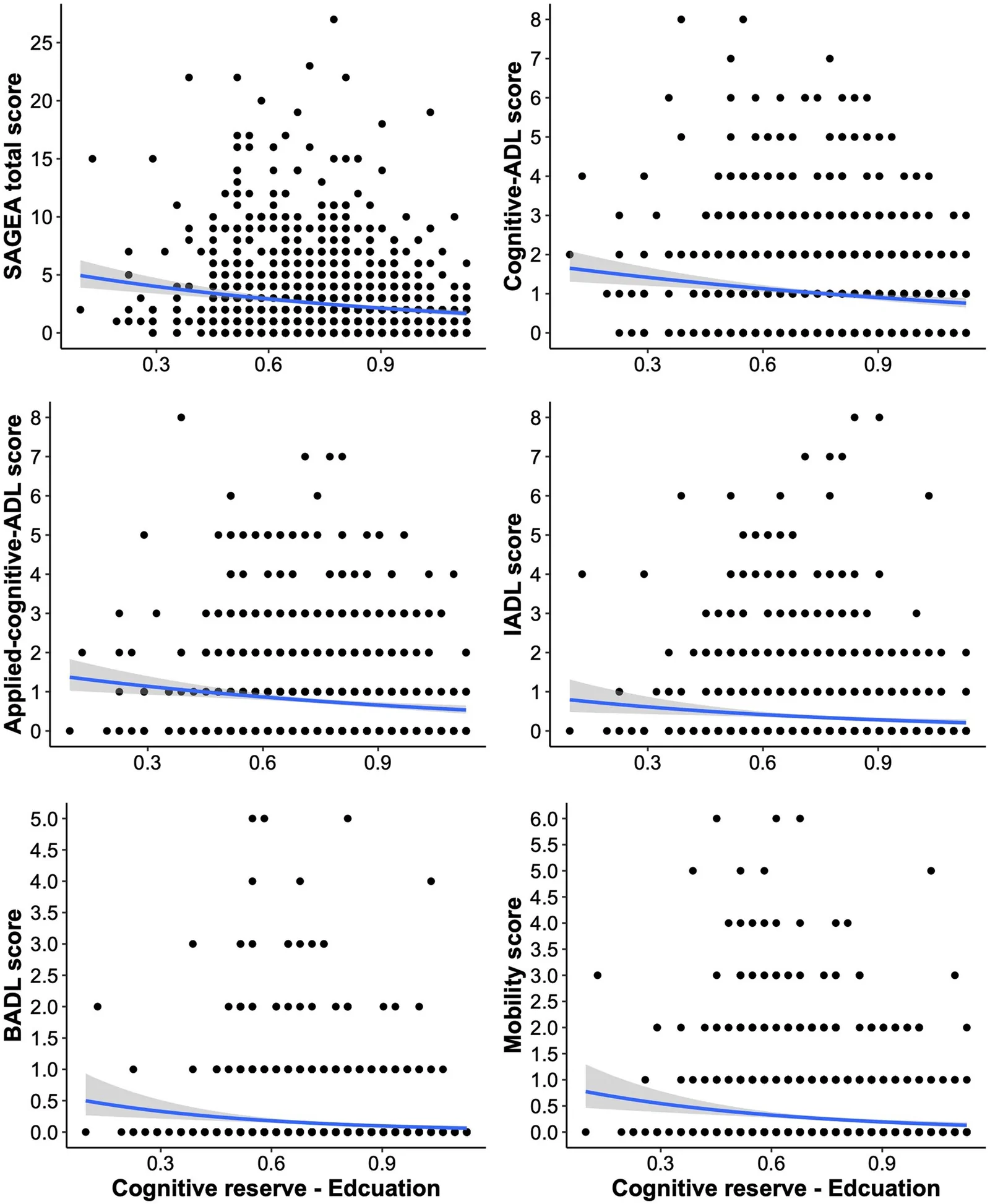
Scatterplots illustrating the associations between the cognitive reserve-education domain and SAGEA total and domain scores. For each plot, negative binomial regression lines are overlaid, with shaded regions representing the corresponding 95% confidence intervals. ADL, activities of daily living; BADL, basic activities of daily living; IADL, instrumental activities of daily living; SAGEA, Standard Assessment of Global Everyday Activities.

**Table 3:** Cross-sectional associations between standardized cognitive reserve score (CRS) and its domains and domain-level ADL performance.

Outcome	Exposure	CR	95% CI	*p*-Value
Cognitive ADL	CRS	0.95	0.90-1.01	.116
	Education	0.94	0.89-1.00	**.046**
	Occupation	1.00	0.95-1.06	.917
	Personal activities	0.96	0.91-1.02	.205
Applied cognitive ADL	CRS	0.93	0.86-1.00	**.038**
	Education	0.93	0.86-1.00	**.041**
	Occupation	0.94	0.87-1.01	.079
	Personal activities	1.00	0.93-1.07	.904
IADL	CRS	0.95	0.84-1.08	.408
	Education	0.89	0.78-1.00	.055
	Occupation	0.97	0.86-1.10	.644
	Personal activities	1.05	0.93-1.19	.434
BADL	CRS	0.89	0.75-1.06	.171
	Education	0.81	0.69-0.96	**.013**
	Occupation	0.90	0.77-1.06	.214
	Personal activities	1.11	0.94-1.30	.219
Mobility	CRS	0.95	0.83-1.08	.438
	Education	0.82	0.72-0.93	**.002**
	Occupation	1.01	0.89-1.15	.852
	Personal activities	1.10	0.97-1.25	.143

Separate models were run for each ADL domain and each cognitive reserve domain. All models were adjusted for age, sex, cognitive performance as z-scores on a neuropsychological test battery, MBI-C total score, and physical/sensory limitations. Bold p-values indicate statistical significance (<0.05).

Abbreviations: ADL, activities of daily living; BADL, basic activities of daily living; CI, confidence interval; CR, count ratio; IADL, instrumental activities of daily living.

### Cognitive reserve, ADL, and QoL: mediation analysis

No significant indirect association was observed between standardized CRS and QoL via global ADL performance (ACME: estimate = 0.009, 95% CI, −0.018 to 0.079, *p* = .202). Likewise, no direct association between cognitive reserve and QoL was observed (ADE: estimate = −0.026, 95% CI, −0.211 to 0.169, *p* = .774); the total effects (*p* = .966) and proportion mediated (0.900) were also non-significant. When the mediation analyses were repeated across cognitive reserve domains, education was the only domain demonstrating significant indirect association with QoL through global ADL performance (ACME: estimate = 0.052, 95% CI, 0.011-0.109, *p* = .014). No significant associations were observed for occupation (ACME: *p* = .788; ADE: *p* = .710) or personal activities (ACME: *p* = .880; ADE: *p* = .908). Nonsignificant total effects and proportions mediated were likewise observed across cognitive reserve domains.

## Discussion

In this cross-sectional study of 1571 CU participants, greater cognitive reserve was associated with better function for both global and domain-specific ADL, which in turn was associated with better QoL. Among cognitive reserve domains, education demonstrated the most robust associations with ADL and QoL, highlighting the potential role of lifelong learning in maintaining functional independence. These findings extend prior evidence linking cognitive reserve to cognitive outcomes by demonstrating that cognitive reserve may also support daily functioning and overall well-being, even before the onset of cognitive impairment.

A substantial body of evidence has established that higher cognitive reserve plays a protective role against cognitive decline in early dementia stages, including normal cognition and MCI. In systematic reviews and meta-analyses, higher cognitive reserve was cross-sectionally and longitudinally associated with better cognitive performance and reduced risk of developing MCI and dementia.^8^^,^^19–21^ Years of education was specifically highlighted to have significant associations with global cognitive test scores.^19^ One study even reported that higher educational attainment was associated with a greater chance of reversion from MCI to normal cognition than progression from MCI to dementia, compared to lower educational attainment.^22^

Fewer studies have focused on the link between cognitive reserve and functional outcomes, but the existing evidence indicates that cognitive reserve may also support independence in daily life. In community-dwelling older adults, cognitive reserve, captured by years of education, moderated the association between global cognition and IADL, such that those with lower education levels were at greater risk for functional decline associated with cognitive decline.^9^ A follow-up study from the same group revealed that cognitive reserve was positively associated with functional ability, with executive functioning mediating this relationship.^23^ Another study of community-dwelling older adults found that cognitive reserve, estimated from pre-morbid intellectual ability, moderated the relationship between cognitive performance and IADL functioning, such that higher cognitive reserve weakened the link between cognitive deficits and functional difficulties.^24^ One study on MCI participants also demonstrated that leisure engagement, as a proxy for cognitive reserve, was strongly associated with better BADL and IADL functioning.^10^

Building on a limited but growing literature, including recent studies linking cognitive reserve to NPS,^12^^,^^25^ our study suggests that the benefits of cognitive reserve extend beyond cognitive performance, also encompassing functional abilities. By identifying specific ADL domains and accounting for cognitive, behavioral, and physical determinants of function, our findings demonstrated that higher cognitive reserve, particularly educational attainment, was associated with lower functional disabilities in CU older adults. Our ADL domain-level analyses further revealed that higher cognitive reserve had the strongest effect on applied cognitive ADL, such as doing concentration-based activities, navigating new environments, and organizing trips or social activities. This finding suggests that cognitive reserve may particularly support complex, cognitively demanding daily activities that require planning, problem-solving, and mental flexibility, rather than routine or habitual tasks. In line with previous studies on the protective effects of cognitive reserve on cognitive performance,^12^^,^^19^^,^^20^ we also identified an association between cognitive reserve and the cognitive ADL domain, which reached significance in a sensitivity analysis excluding adjustment for neuropsychological test scores. This finding suggests that controlling for cognitive performance in models with cognitive ADL as the outcome, may partially attenuate the relationship with cognitive reserve, likely due to overlap between cognitive ADL tasks and certain domains assessed by neuropsychological testing. However, while related, cognitive ADL and neuropsychological tests capture distinct aspects of cognitive ability. Cognitive ADL tasks reflect real-world cognitive demands that, beyond test-based abilities, require the flexible integration of multiple processes in dynamic, unstructured, and multitasking daily contexts. Consequently, cognitive ADL provides an ecologically valid measure of how cognitive abilities are translated into everyday functional independence, complementing rather than duplicating information obtained from standardized neuropsychological assessments.

Interestingly, when cognitive reserve was decomposed into its domains, higher education was the only domain consistently associated with better performance across all ADL domains, with particularly strong effects on BADL and mobility. Importantly, the association with BADL and mobility persisted even after adjusting for physical/sensory limitations, underscoring the broad influence of education not only on cognitively demanding tasks but also on more basic self-care and physical functioning. While education is a core proxy of cognitive reserve, it also reflects broader life-course advantages that extend beyond formal schooling. Higher educational attainment is closely associated with socioeconomic status, health literacy, healthier lifestyle behaviors, and greater access to healthcare and other health-promoting resources. Collectively, these factors may contribute to the preservation of everyday functioning through multiple, complementary pathways and may partly explain the strong associations of cognitive reserve with both BADL and mobility domains. From a cognitive perspective, higher educational attainment may foster more effective problem-solving, planning, and cognitive flexibility, enabling individuals to develop adaptive strategies that preserve functional independence despite age-related cognitive or physical limitations. For example, individuals with greater cognitive reserve may be better equipped to recognize functional decline, seek timely healthcare, adhere to treatment recommendations, or effectively use assistive devices (eg, walking aids) and environmental modifications to compensate for emerging impairments. At the same time, greater health literacy may facilitate proactive management of chronic conditions, engagement in preventive health behaviors, and maintenance of physical health, all of which are important determinants of both basic and instrumental functional abilities. Together, these interpretations support a life-course perspective in which educational attainment serves not only as a marker of cognitive reserve but also as an indicator of accumulated biological, cognitive, and social advantages that promote resilience and functional independence in later life. This interpretation is further supported by the evidence suggesting that early-life experiences, such as formal education, may provide stronger protection against future cognitive, behavioral, and functional changes than later-life activities.^12^^,^^26^^,^^27^ This evidence may also help explain the lack of significant associations with the cognitive reserve domains of occupation and personal activities in our study. It is also possible that educational attainment represents a more stable, cumulative marker of lifelong cognitive enrichment than occupation or later-life leisure activities. The latter may fluctuate over time and be more susceptible to changes in health status. It is still important to note that the occupation domain in our study showed a trend toward significance specifically with the applied cognitive ADL domain. The observed trend further highlights the importance of cognitive reserve obtained from occupational complexity for the high-demand cognitive activities involved in the applied cognitive ADL. The lack of significant associations between personal leisure activities (musical instrument, multilingualism, engagement in cognitively stimulating activities) and ADL in our study contrasts with a few previous reports of significant associations. In a longitudinal study of community-dwelling Chinese older adults, leisure activities captured through reading books or newspapers were associated with a lower risk of IADL impairment.^28^ This discrepancy may reflect differences in the leisure activities examined. Reading is typically a frequent, ongoing activity that may provide sustained cognitive stimulation over time, whereas activities such as playing a musical instrument or completing puzzles may occur less regularly. Moreover, multilingualism and musical ability represent lifetime experiences that may not reflect current engagement, as individuals may have acquired these skills many years earlier without continuing to actively use them. Consequently, reading habits may better capture the cumulative effects of ongoing cognitive engagement that are more relevant to maintaining functional independence. Similarly, a three-year Japanese study of community-dwelling older adults reported that engagement in more leisure activities was associated with lower IADL impairment, compared to fewer leisure activities.^29^ However, compared to our work, this study assessed a much broader range of physical, cultural, social, and recreational leisure activities. Such comprehensive measures may better capture the diversity, frequency, and cumulative effects of leisure engagement that contribute to maintaining functional independence. It is also important to note that, as opposed to our study, both cited studies employed a longitudinal design, enabling the assessment of future functional decline. Replication of our findings using longitudinal data may provide additional insight into whether the leisure component of cognitive reserve contributes to the preservation of ADL over time, even if cross-sectional associations are not apparent.

Beyond cognitive reserve, a few other model covariates in our study also showed significant associations with ADL performance. Higher neuropsychological test scores were associated with lower total ADL scores, in line with the literature indicating a link between cognition and functional independence. Conversely, higher MBI-C total scores and greater physical/sensory limitations were associated with higher total ADL scores, reflecting greater functional impairment. These results are in line with the literature,^30^^,^^31^ highlighting that, alongside cognitive reserve, both cognitive and mental and physical health are important determinants of functional ability, emphasizing the multifactorial nature of functional independence in daily life.

The mediation analysis reflecting indirect associations between the education domain of cognitive reserve and QoL via functional ability in our study reinforces the potential importance of maintaining autonomy in daily activities for overall well-being. These findings align with the population-based literature linking cognitive reserve with higher QoL.^32^ Within the hypothesized framework examined in this study, even CU individuals with higher educational attainment appeared to have better functional abilities, which were in turn associated with life satisfaction and enhanced social relationships. This finding suggests that education-related aspects of cognitive reserve may contribute to quality of life, at least in part, through preservation of functional independence. However, given the cross-sectional design, these findings should be interpreted as describing patterns of association rather than confirming a temporal or causal pathway. Longitudinal follow-up studies will be important to determine causal relationships over time.

This study has several limitations. Socioeconomic and sociocultural factors, such as differences in access to education, opportunities for higher-level cognitively demanding occupations, and engagement in leisure activities, may have influenced both cognitive reserve and functional outcomes,^33^ potentially confounding the observed associations. In addition, cultural factors, including historical and gender-related differences in educational and occupational opportunities, may shape the development of cognitive reserve, particularly among individuals from generations or communities with unequal access to formal education and cognitively stimulating experiences. The CAN-PROTECT cohort is predominantly female, which may limit the generalizability of findings to populations with more balanced sex distributions. Furthermore, recruitment through an online platform may introduce selection bias, as individuals who enroll in digital research cohorts may differ from the broader population in terms of access to technology, health engagement, and baseline cognitive or functional status. These factors may have contributed to a sample with relatively high cognitive and functional abilities, which should be considered when interpreting the findings. Our cross-sectional study design precludes causal inference, and reliance on self-reported ADL measures may be subject to reporting bias. Further, the relationship between cognitive reserve and ADL may be bidirectional: while higher cognitive reserve may support better functional performance, engagement in cognitively demanding activities captured by ADL could also enhance cognitive reserve. With the ongoing longitudinal follow-up of CAN-PROTECT, future longitudinal studies could help clarify these limitations. Specifically, future analyses could evaluate whether baseline cognitive reserve predicts subsequent changes in ADL and QoL, and whether changes in functional abilities and cognitively stimulating activities are associated with subsequent trajectories of cognitive reserve-related outcomes. These longitudinal approaches will help clarify the directionality of associations and determine whether cognitive reserve contributes to resilience against functional decline over time. CAN-PROTECT also collects study partner-reported ADL data, though the current sample size is limited. Future analyses could leverage these reports as the cohort expands to replicate our findings. Another potential limitation is the possibility of circularity between cognitive reserve and ADL. One component of cognitive reserve in our study includes engagement in cognitively stimulating activities, such as word or number puzzles. At the same time, the applied cognitive ADL domain of the SAGEA scale also captures abilities related to playing games. This overlap could potentially inflate associations between cognitive reserve and ADL. However, reassuringly, sensitivity analyses excluding the concentration-based games item from the applied cognitive ADL domain yielded comparable results in association with CRS (SAGEA total score: CR = 0.94, 95% CI, 0.89-1.00, *p* = .039), suggesting that the association between cognitive reserve and ADL was not driven by this specific overlapping activity. Furthermore, domain-level analyses examining the individual components of cognitive reserve provided evidence that the association was primarily driven by educational attainment rather than the occupation and personal activities domains. Together, these findings suggest that the observed relationships reflect broader aspects of cognitive reserve rather than being solely attributable to overlap between cognitively stimulating leisure activities and ADL measurement. Further, our operationalization of cognitive reserve may not capture other potential proxy measures of cognitive reserve. Similarly, while we accounted for certain physical and sensory conditions potentially contributing to ADL limitations independent of underlying neurodegeneration, other comorbid medical conditions, not captured in our study, may also influence function. Future research incorporating comprehensive measures of comorbidity would help clarify the relative contributions of non-cognitive factors to early functional decline.

Further research is needed to clarify the neural mechanisms through which cognitive reserve supports the preservation of daily functioning in the early stages of neurodegenerative disease. Growing neuroimaging evidence suggests that cognitive reserve may promote resilience through both neural efficiency, characterized by more efficient recruitment of brain networks during cognitive tasks, and neural compensation, whereby alternative neural circuits are engaged as cognitive demands increase or neuropathology accumulates. Consistent with this framework, functional magnetic resonance imaging (MRI) studies have linked neural efficiency to the integrity of the default mode network and medial temporal regions. Compensatory processes have been associated with greater recruitment of frontal regions and the dorsal attention network, particularly in individuals with MCI and AD.^34^ Greater efficiency of the default mode network has been associated with higher cognitive reserve.^35^^,^^36^ This association suggests a potential neural basis for preserving cognitive processes underlying complex everyday functional abilities, including memory, attention, and executive control. Emerging evidence also implicates the locus coeruleus-noradrenergic system as a potential contributor to cognitive reserve. Findings suggest that individual differences in reserve may moderate the relationship between locus coeruleus connectivity and cognitive performance in memory and executive domains.^37^ Importantly, these cognitive systems are also central to the performance of instrumental ADL, suggesting a plausible pathway through which reserve-related neural mechanisms may translate into preserved real-world functioning. In line with this evidence, another functional MRI study also reported that locus coeruleus volume predicted higher attentional performance as well as biological brain maintenance across the cognitive spectrum.^38^ Importantly, converging evidence indicates that the moderating effects of cognitive reserve may extend beyond cognition to other clinical manifestations of neurodegenerative disease. In a recent study on a sample spanning CU, MCI, and dementia, higher cognitive reserve attenuated the association between hippocampal atrophy and neuropsychiatric symptom burden.^25^ This finding suggests that cognitive reserve may buffer the clinical expression of underlying brain pathology beyond cognitive outcomes. Similarly, another study on a large sample of CU participants reported a link between higher cognitive reserve and lower NPS severity.^12^ Taken together, these findings support the view that cognitive reserve may influence how neurodegeneration translates into multiple clinical phenotypes, including cognition, behavior, and potentially everyday functional abilities. More broadly, these findings align with contemporary geroscience perspectives that conceptualize resilience as a systems-level property. This perspective emphasizes interactions among neural networks, neuromodulatory pathways, vascular and glial processes, and other biological mechanisms that may preserve function despite age-related stressors.^39^^,^^40^ The present study was not designed to examine neural and biological mechanisms underlying the observed associations between cognitive reserve, everyday functioning, and quality of life. Future longitudinal studies integrating multimodal neuroimaging and functional assessments will be essential to determine whether such neural and systems-level resilience processes contribute to these associations in CU older adults.

Nonetheless, our findings underscore the potential value of interventions aimed at enhancing cognitive reserve to promote functional independence and QoL in aging populations. Encouraging engagement in applied cognitive tasks, social organization, and complex problem-solving may help preserve functional capacity, life satisfaction, and social relationships, even before cognitive impairment emerges. Importantly, our results also suggest that the benefits of higher cognitive reserve extend beyond complex activities to include basic self-care, highlighting the broad relevance of cognitive reserve for supporting independence across the full spectrum of daily functioning.

## Data Availability

Due to the confidential nature of participant demographic health data, CAN-PROTECT study data are not publicly available in a data repository. However, individual access to the data may be provided upon reasonable request to the corresponding author.
